# Two‐drug antiretroviral regimens: an assessment of virologic response and durability among treatment‐experienced persons living with HIV in the OPERA^®^ Observational Database

**DOI:** 10.1002/jia2.25418

**Published:** 2019-12-04

**Authors:** Gerald Pierone, Cassidy Henegar, Jennifer Fusco, Vani Vannappagari, Michael Aboud, Leigh Ragone, Gregory Fusco

**Affiliations:** ^1^ Whole Family Health Center Vero Beach FL USA; ^2^ ViiV Healthcare Research Triangle Park NC USA; ^3^ Epividian, Inc Durham NC USA; ^4^ ViiV Healthcare London United Kingdom

**Keywords:** HIV, human immunodeficiency virus, ART‐experience, antiretroviral therapy‐experience, regimens, two‐drug, cohort

## Abstract

**Introduction:**

Two‐drug regimens (2‐DR) have the potential to be a viable solution to the challenges of treatment complexity, cost, adverse effects and contraindications. We sought to describe the real‐world use and effectiveness of 2‐DR among persons living with HIV (PLHIV) in the United States.

**Methods:**

We analysed data for 10,190 treatment‐experienced patients from the OPERA® Observational Database initiating a new 2‐DR or three‐drug regimen (3‐DR) between 1 January 2010 and 30 June 2016. Multivariate Cox Proportional Hazards models were used to estimate the association among 2‐DR or 3‐DR initiation and virologic suppression (viral load (VL) <50 copies/mL), virologic failure (2 VLs > 200 copies/mL or 1 VL > 200 copies/mL + discontinuation) or regimen discontinuation.

**Results:**

Patients initiating a 2‐DR (n = 1337, 13%) were older, and more likely to have a lower CD4 count, a history of AIDS and comorbid conditions than patients initiating a 3‐DR. There was no difference between groups in time to virologic suppression (aHR: 1.00 (95% CI: 0.88, 1.13)) among viraemic patients (baseline VL ≥ 50 copies/mL, n = 4180), or time to virologic failure (aHR: 1.15 (95% CI: 0.90, 1.48)) among virologically stable patients (baseline VL < 50 copies/mL, n = 6010). However, time to discontinuation was shorter following 2‐DR than 3‐DR initiation (aHR: 1.51 (95% CI: 1.41, 1.61)).

**Conclusions:**

In this large cohort of treatment‐experienced patients, 2‐DR prescriptions were common and more frequent among patients with significant comorbidity. Virologic response was similar, but duration of use was shorter with a 2‐DR than a 3‐DR, suggesting that 2‐DRs may be a virologically effective treatment strategy for treatment‐experienced PLHIV with existing comorbidities.

## 1 Introduction

Antiretroviral (ARV) drugs are potent and effective; however, there are toxicity concerns with multi‐agent regimens, especially for those containing nucleos(t)ide reverse transcriptase inhibitors (NRTI) or protease inhibitors (PI) 1, 2, 3, 4. For therapeutically complex patients, drug‐sparing regimens such as two‐drug regimens (2‐DR) that are not pharmacokinetically enhanced have the potential to reduce treatment complexity, cost, adverse side effects and contraindications (e.g. hepatitis C virus (HCV) therapy, anti‐diabetics, statins) 5, 6. As persons living with HIV (PLHIV) continue to age, increasing comorbidity results in higher poly‐pharmacy, and these additional complexities can interfere with chosen therapeutic strategies and patient adherence 7, 8. Regimen simplification, including reductions in the overall pill burden and dosing frequency, can also improve patient adherence 9. Thus, there has been renewed interest in exploring 2‐DRs as a viable solution to these challenges.

There is a growing body of clinical trial evidence that 2‐DRs may be effective in maintaining virologic suppression among treatment‐experienced patients 4, 10, 11, 12, 13. 2‐DRs containing ritonavir‐boosted PIs or integrase strand transfer inhibitors (INSTI) have been most promising 13. Lopinavir/ritonavir (LPV/r) + lamivudine (3TC) demonstrated comparable efficacy and tolerability at 48 weeks in the OLE study 14. The dual combination of atazanavir (ATV)/r + 3TC has also been evaluated among suppressed, treatment‐experienced patients in the AtLaS‐M 15 and SALT 16 studies. In both trials, maintenance of virologic efficacy, defined as a viral load (VL) <50 copies/mL, was demonstrated at 96 and 24 weeks respectively 15, 16. The DUAL‐GESIDA trial demonstrated similar efficacy and tolerability after 48 weeks for the 2‐DR regimen containing darunavir (DRV)/r + 3TC 17.

Treatment simplification to a 2‐DR containing a PI and raltegravir (RAL) was assessed in two small trials and also showed comparable efficacy when evaluated against either 3‐DRs 18 or a standard regimen containing at least two NRTIs 19, although in a third trial, the only two virologic failures occurred in the ATV/r + RAL arm 20. The RALAM trial, compared RAL + 3TC to standard 3‐DR in experienced, suppressed individuals without a history of virologic failure or hepatitis B (HBV) infection and recorded no virologic failures or blips through 24 weeks 21.

Recent clinical trials have evaluated the efficacy of 2‐DRs containing dolutegravir (DTG) + rilpivirine (RPV) 22, 23, DTG + 3TC 24, 25 and cabotegravir + RPV 26. In a pooled analysis of two open‐label multicentre phase III clinical trials of suppressed, treatment‐experienced patients, SWORD 1 and SWORD 2, DTG + RPV was non‐inferior to 3‐DRs and four‐drug regimens for virologic suppression maintenance (VL < 50 copies/mL at 48 weeks) 22 and a low rate of virologic failures at week 100 in PLHIV who switched to DTG + RPV at randomization or at week 52 23. In November 2017, this co‐formulation became the first complete treatment regimen containing only two ARV drugs to be approved by the US Food and Drug Administration 27.

An observational study reported that 93% of the patients who switched from a 3‐DR to DRV/r + RAL had an undetectable VL at 48 weeks 28. Another evaluated those who switched to DTG + RPV or DTG + 3TC for virologic failure and treatment discontinuation. Few failures were observed and a high probability of remaining on the regimen in both groups 29. In a large cohort study of treatment experienced PLHIV initiating a variety of 2‐DRs compared to 3‐DRs with the same core agents saw no difference in the proportion with controlled viraemia (<400 copies) or treatment failures (>400 copies, regimen change, adverse event or death) at 6 or 12 months 30.

Several systematic reviews and meta‐analyses of trials assessing the efficacy and safety of 2‐DRs have found comparable efficacy to standard 3‐DRs, especially among suppressed, treatment‐experienced patients with PI‐and INSTI‐based regimens 10, 11, 12, 13. Punekar *et al*. performed a systematic review and meta‐analysis of real‐world data of experienced, suppressed patients predominantly in Europe. Virologic suppression (<50 copies), virologic failure and discontinuation at 48 weeks post switch to DTG + 3TC or DTG + RPV demonstrating effectiveness and durability 31.

We sought to compare treatment‐experienced patients initiating 2‐DRs to treatment‐experienced patients initiating 3‐DRs in a large, clinical cohort of PLHIV in the US by demographic and clinical characteristics as well as treatment outcomes over time: virologic suppression, virologic failure and regimen discontinuation.

## 2 Methods

### 2.1 Study sample

The OPERA^®^ Observational Database, a prospective cohort of 79803 PLHIV treated at 79 outpatient clinical sites in the United States was used for this study and includes comprehensive patient‐level information from electronic health records, including diagnoses, clinical history, medications, laboratory results and demographic information. The OPERA database obtains annual institutional review board (IRB) approval from Advarra IRB, including a waiver of informed consent and authorization for the use of protected health information.

Treatment‐experienced PLHIV who initiated a new 2‐DR or 3‐DR between 1 January 2010 and 30 June 2016 were identified. The period of follow‐up extended until 30 June 2017 to allow patients the potential for at least a year of follow‐up. There were 33560 patients who initiated an eligible 2‐DR or 3‐DR, who had a clinic visit within seven days of the start date of the regimen of interest, and had a VL within 120 days prior to regimen initiation. When the population was limited to those who were treatment‐experienced and whose regimen of interest was not part of a clinical trial, the final analysis population totalled 10190 patients.

Patients were considered lost to follow‐up if they had not had contact with the clinic in more than a year. In the absence of an event, patients were censored at death, loss to follow‐up or the end of the follow‐up period (30 June 2017). For analyses with virologic suppression and virologic failure as the outcome, patients were also censored at regimen discontinuation.

### 2.2 Regimen type

Regimens of interest consisted of a 2‐DR or 3‐DR initiated between 1 January 2010 and 30 June 2016. Regimens had to be at least 30 days in duration and initiated after the patient's first active visit in OPERA. The first 2‐DR initiated by a patient during this period was used for analysis. For patients who did not initiate a 2‐DR, the first 3‐DR initiated during this period was used for analysis. All regimens had to contain at least one core agent (PI, non‐nucleoside reverse transcriptase inhibitor (NNRTI), or INSTI). Boosting agents did not contribute to the ARV number when identifying regimens of interest.

### 2.3 Stratification

The study population was stratified based on baseline VL. Patients were considered viraemic if their last VL before or at baseline was ≥50 copies/mL. Patients were considered virologically stable if their last VL before or at baseline was <50 copies/mL.

### 2.4 Virologic outcomes

The virologic outcomes included virologic suppression, virologic failure and regimen discontinuation. Virologic suppression was assessed in patients who were viraemic and defined as achieving a VL < 50 copies/mL on the regimen of interest. Among patients who were virologically stable, the primary outcome was virologic failure, defined as two consecutive VLs > 200 copies/mL over the period of follow‐up, or one VL > 200 copies/mL followed by discontinuation of the regimen. Discontinuation was assessed among all patients and was defined as stopping or changing any component of the regimen of interest.

### 2.5 Covariates

Baseline demographic and clinical characteristics were defined as characteristics measured on the date a 2‐DR regimen or 3‐DR regimen was initiated. If any values were missing at the time of initiation, the last reported value prior to baseline was used.

### 2.6 Statistical analysis

Categorical variables were compared between 2‐DR and 3‐DR using the Pearson chi‐square test or Fishers exact test, as applicable, and continuous variables were compared using the Wilcoxon rank‐sum test. Kaplan‐Meier survival curves were used to estimate time to virologic suppression, time to virologic failure and time to discontinuation by regimen type. The log‐rank test was used to compare unadjusted differences between patients initiating 2‐DR and 3‐DR in time to each virologic outcome. Univariable and multivariable Cox proportional hazards regression models were used to evaluate the association between regimen type and each outcome. Unadjusted and adjusted hazard ratios (HR and aHR) with 95% confidence intervals (CI) were reported using 3‐DR as the referent group. For adjusted analyses, confounders were selected a priori, using a directed acyclic graph (DAGs). All models were adjusted for sex, race, age, substance abuse, comorbidity (diagnoses of cardiovascular disease, endocrine disorders, liver disease, renal disease, peripheral neuropathy or mental health disorder), prior lines of antiretroviral therapy (ART) and total time on ART. Additional covariates were included in the virologic suppression model (ADAP/Ryan White programme participation and baseline VL), the virologic failure model (baseline CD4 cell count), and the discontinuation model (ADAP/Ryan White programme participation, baseline VL, baseline CD4 cell count and 3‐DR use immediately prior to baseline). The proportional hazards assumption was assessed graphically by plotting the log of the cumulative hazard over time. Additional sensitivity analyses were conducted comparing time to discontinuation stratified by virologic status at baseline (viraemic, virologically stable).

## 3 Results

### 3.1 Study population

**Figure 1:**
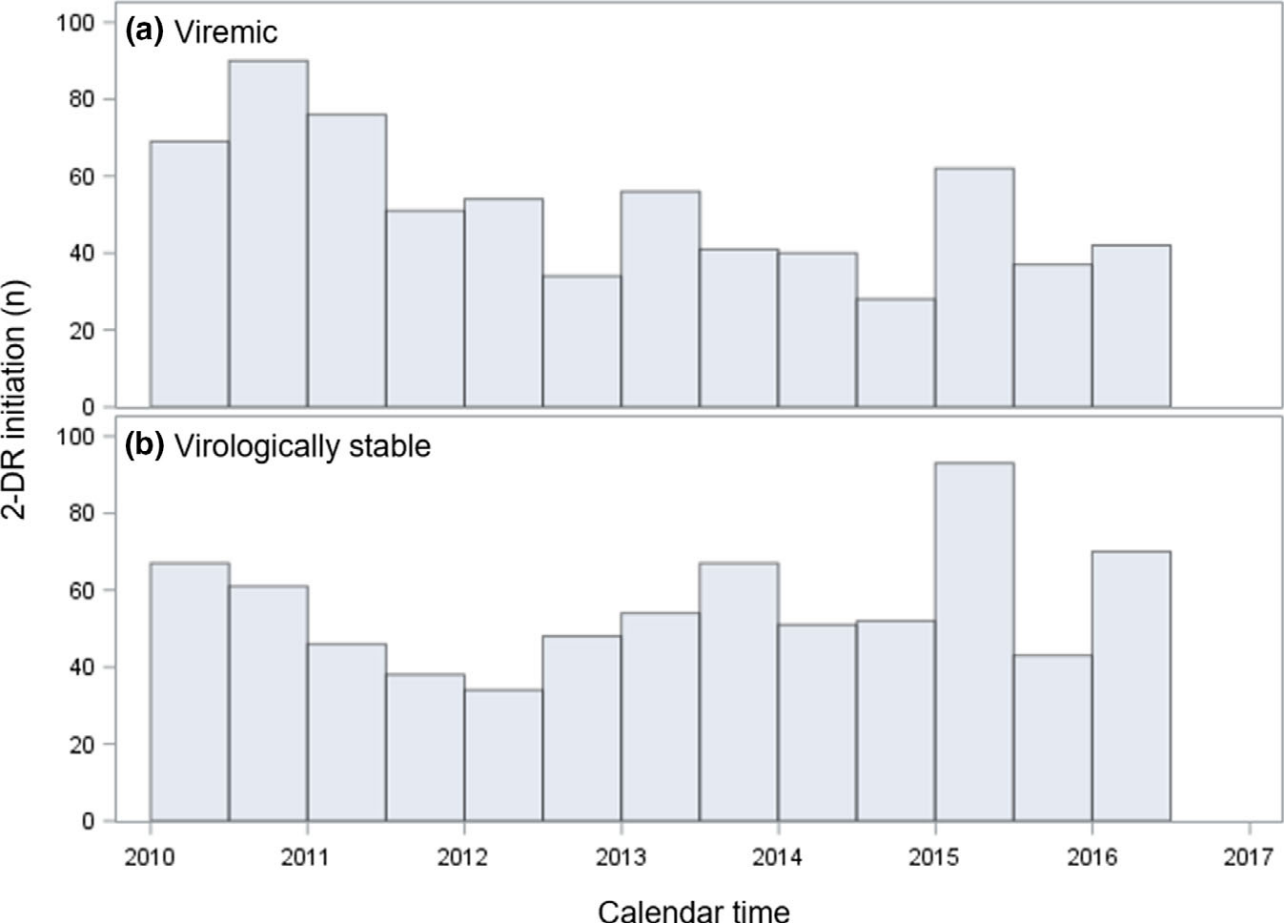
Calendar year of 2‐drug regimen initiation among (a) patients viraemic at baseline and (b) patients virologically stable at baseline.

In the overall sample of 10190 treatment‐experienced patients, 13% (n = 1337) initiated a 2‐DR and 87% (n = 8853) initiated a 3‐DR between 1 January 2010 and 30 June 2016. Baseline demographic and clinical characteristics varied by group (Table 1). The median age was 50 years (IQR: 44, 57) among patients who initiated a 2‐DR, and 46 years (IQR: 40, 53) among patients who initiated a 3‐DR. Compared to patients on 3‐DRs, patients on 2‐DRs were more likely to be female, African‐American and have received Medicare or Medicaid, but they were less likely to have received ADAP/Ryan White. With respect to clinical characteristics, patients on 2‐DRs had a longer duration of ART and were more likely to have received at least five lines of ART prior to the current regimen of interest. Patients on 2‐DRs were also more likely to have a history of AIDS and other comorbidities. Initiation of 2‐DRs occurred throughout the observation period, without any substantial peak in 2‐DR prescription (Figure 1).

**Table 1:** Baseline demographic and clinical characteristics of treatment‐experienced patients initiating 2‐DR or 3‐DR in the OPERA cohort between 1 January 2010 and 30 June 2016 (n = 10190)

Characteristic	2‐DR regimens (n = 1337)		3‐DR regimens (n = 8853)		*p*‐values
	n	%	n	%	
Demographic					
Age (years)^a^	50 (44, 57)		46 (39, 53)		<.0001
Female sex	296	22.1	1441	16.3	<.0001
African‐American	509	38.1	2544	28.7	<.0001
Hispanic ethnicity	268	20.0	2326	26.3	<.0001
Men who have sex with men	542	40.5	4742	53.6	<.0001
Substance Abuse	251	18.8	1831	20.7	0.1066
Region: South	813	60.8	4031	45.5	<.0001
Medicaid	369	27.6	2106	23.8	0.0025
Medicare	342	25.6	1336	15.1	<.0001
ADAP/Ryan White	297	22.2	2610	29.5	<.0001
Clinical					
≥5 prior lines of ART	558	41.7	1773	20.0	<.0001
On 3‐DR immediately prior to baseline	650	48.6	3184	36.0	<.0001
Time since ART initiation (months)^a^	60 (20, 117)		46 (17, 98)		<.0001
HIV RNA < 50 copies/mL	724	54.2	5286	59.7	<.0001
Baseline CD4> 500 cells/μL	528	39.5	4367	49.3	<.0001
History of AIDS‐defining event	569	42.6	2401	27.1	<.0001
VACS score^a^, ^b^	27 (13, 43)		17 (6, 28)		<.0001
Cardiovascular disease	282	21.1	943	10.7	<.0001
Endocrine disorders	757	56.6	4082	46.1	<.0001
Liver disease	353	26.4	2034	23.0	0.0058
Peripheral neuropathy	305	22.8	1175	13.3	<.0001
Renal disease	437	32.7	840	9.5	<.0001
Hypertension	635	47.5	2761	31.2	<.0001

a Median (IQR)

b VACS Mortality Index: Scored by summing pre‐assigned points for age, CD4 count, HIV‐1 RNA, haemoglobin, platelets, aspartate and alanine transaminase, creatinine and viral hepatitis C infection. A higher score is associated with a higher risk of 5‐year all‐cause mortality.

Median baseline VL in patients viraemic at 2‐DR initiation was 3759 (IQR: 223, 48201) copies/mL; median log^10^ VL was 3.6 (IQR: 2.3, 4.7). Median VL in patients viraemic at 3‐DR initiation was 4900 (IQR: 170, 58200) copies/mL; median log^10^ VL was 3.7 (IQR: 2.2, 4.8). These were not statistically significantly different (*p* = 0.9406).

**Figure 2:**
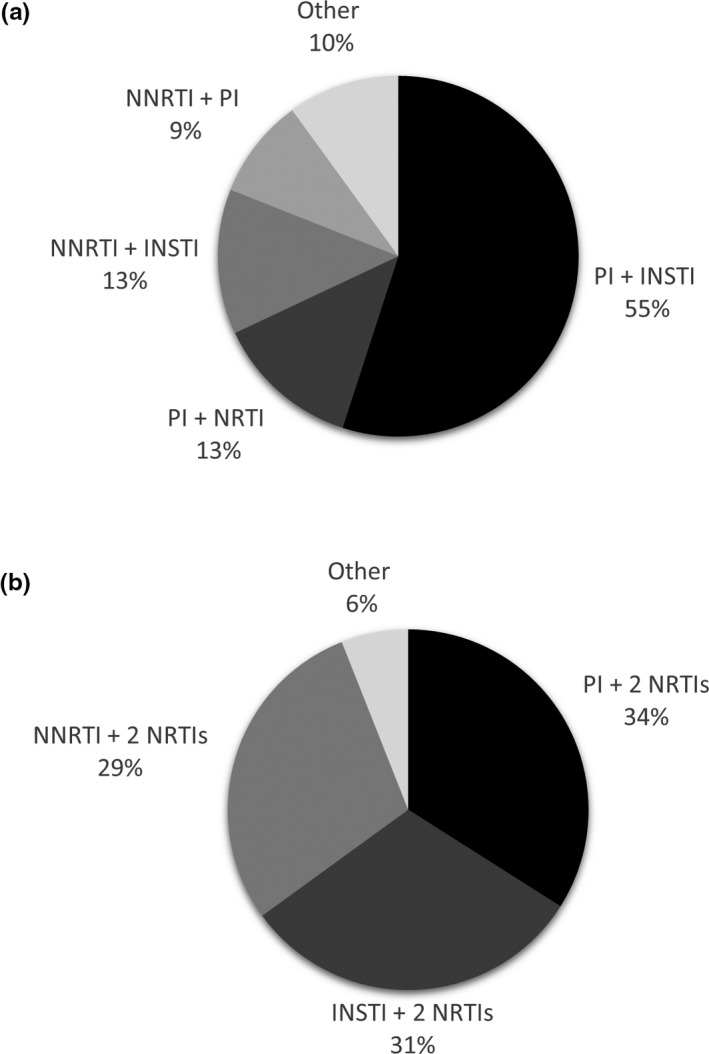
Most common (a) 2‐DR regimens and (b) 3‐DR regimens initiated among treatment‐experienced patients in the OPERA cohort between 1 January 2010 and 30 June 2016. (n = 10190).

As shown in Figure 2, the most common 2‐DRs consisted of a PI and an INSTI (55%), followed by a PI and an NRTI (13%), or an NNRTI and an INSTI (13%). More specifically, patients initiating a 2‐DR most commonly used DRV + RAL (28%), DRV + DTG (16%) or etravirine (ETV) + RAL (7%). The most common 3‐DRs contained two NRTIs paired with either a PI (34%), an INSTI (31%), or an NNRTI (29%). Patients initiating a 3‐DR most commonly used efavirenz (EFV) + emtricitabine (FTC) + tenofovir (TDF) (15%), DRV + FTC+ TDF (12%) or DTG + abacavir (ABC) + 3TC (10%).

### 3.2 Virologic suppression

**Figure 3:**
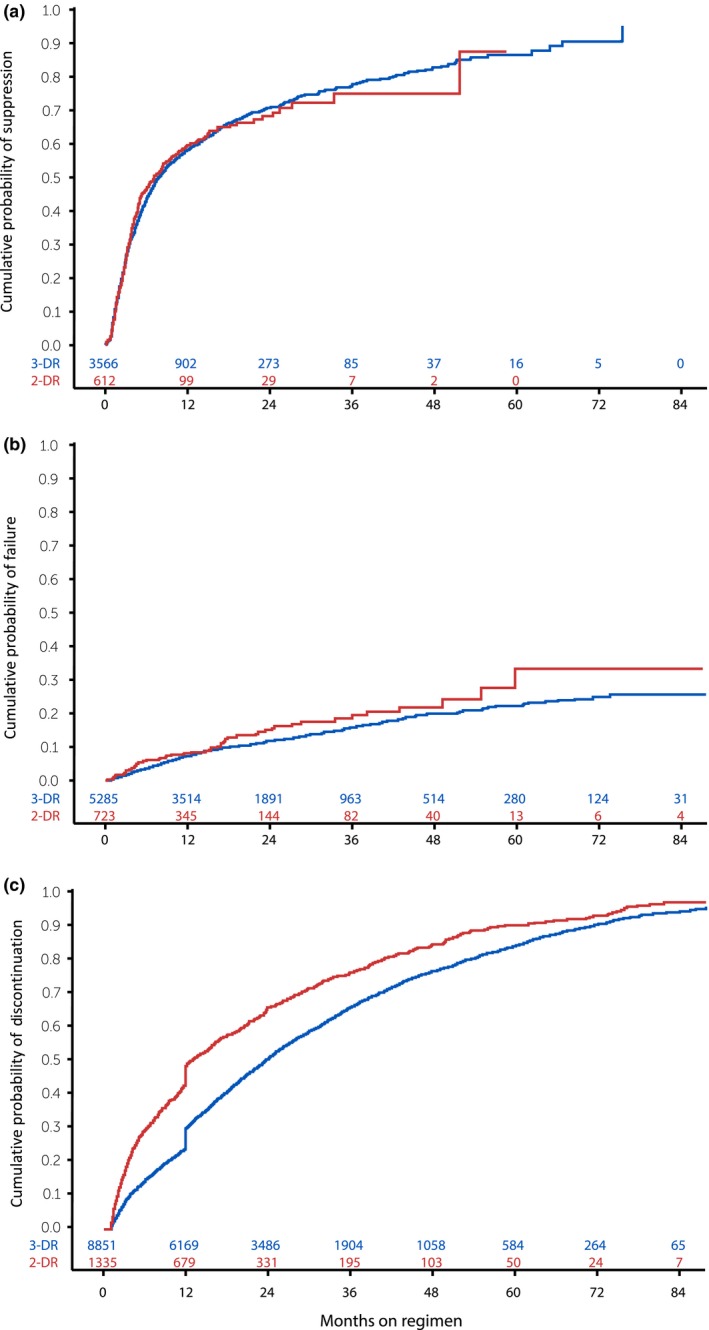
Kaplan‐Meier estimation of the cumulative probability of (a) suppression^†^, (b) failure^‡^, and (c) discontinuation^§^ by regimen type. ^†^Among patients viraemic at baseline (n = 4178). ^‡^Among patients virologically stable at baseline (n = 6008). ^§^Among all patients (n = 10190).

Among 4180 patients who were viraemic at baseline, the virologic suppression incidence rate was 86.8 per 100 person‐years (95% CI: 77.7, 96.8) among patients on a 2‐DR. In contrast, the suppression rate was 78.2 per 100 person‐year (95% CI: 74.9, 81.6) among patients on a 3‐DR (Table 2). Time to virologic suppression did not differ for those who initiated a 2‐DR compared to patients who initiated a 3‐DR (*p* = 0.71, Figure 3a). The adjusted hazard ratio for time to virologic suppression was 1.00 (95% CI: 0.88, 1.13, Table 2).

**Table 2:** Unadjusted and adjusted hazard ratio for time to virologic suppression, virologic failure and discontinuation, comparing patients initiating 2‐DR regimens to patients initiating 3‐DR regimens in the OPERA cohort (n = 10190)

	No. of events	Person‐years	Event rate per 100 patient‐years (95% CI)	Unadjusted HR (95% CI)	Adjusted HR^a^ (95% CI)
Time to virologic suppression^b^: patients viraemic at baseline					
3‐DR	2116	2695.7	78.2 (74.9, 81.6)	1	1
2‐DR	318	366.4	86.8 (77.7, 96.8)	1.02 (0.91, 1.15)	1.00 (0.88, 1.13)
Time to virologic failure^c^: patients virologically stable at baseline					
3‐DR	589	9840.0	6.0 (5.5, 6.5)	1	1
2‐DR	74	936.7	7.9 (6.3, 9.9)	1.26 (0.99, 1.61)	1.15 (0.90, 1.48)
Time to discontinuation^d^: all patients					
3‐DR	6269	17785.2	35.2 (34.4, 36.1)	1	1
2‐DR	1029	1992.0	51.6 (48.6, 54.9)	1.47 (1.38, 1.58)	1.51 (1.41, 1.61)

2‐DR, 2‐drug; 3‐DR, 3‐drug; HR, hazard ratio; CI, confidence interval.

a All multivariable Cox models were adjusted for sex, race, age, substance abuse, comorbidity diagnoses, prior lines of ART and total time on ART

b virologic suppression model was further adjusted for ADAP/Ryan White programme participation, and baseline viral load

c virologic failure model was further adjusted for baseline CD4

d discontinuation model was further adjusted for ADAP/Ryan White programme participation, baseline viral load, baseline CD4 and prior regimen type.

### 3.3 Virologic failure

Among 6010 patients who were virologically stable at baseline, the incidence rate of virologic failure was 7.9 per 100 person‐years (95% CI: 6.3, 9.9) among patients on a 2‐DR and 6.0 per 100 person‐years (95% CI: 5.5, 6.5) among patients on a 3‐DR (Table 2). The difference in time to virologic failure was marginal between groups (*p* = 0.06, Figure 3b). The adjusted hazard ratio for time to virologic failure was 1.15 (95% CI: 0.90, 1.48, Table 2).

### 3.4 Regimen discontinuation

Over follow‐up, the incidence rate of regimen discontinuation was 51.6 per 100 person‐years (95% CI: 48.6, 54.9) among patients on a 2‐DR and 35.2 per 100 person‐years (95% CI: 34.4, 36.1) among patients on a 3‐DR regimen (Table 2). Furthermore, patients who initiated a 2‐DR discontinued more quickly than patients who initiated a 3‐DR (Figure 3c). After adjustment for confounding, time to discontinuation was 1.51 times faster for patients who initiated a 2‐DR regimen than patients who initiated a 3‐DR regimen (95% CI: 1.41, 1.61, Table 2). These findings were consistent regardless of whether patients were viraemic or virologically stable at baseline (data not shown).

## 4 Discussion

Our findings suggest that a sizeable proportion (13%) of treatment‐experienced patients were being treated with a 2‐DR, despite the absence of 2‐DR‐recommended regimens during the study period. More than half of all patients initiating a 2‐DR were on a combination of a PI and INSTI. The two most common specific 2‐DR were DRV + RAL and DRV + DTG, but we observed a variety of specific 2‐DRs and 3‐DRs among patients. In both adjusted and unadjusted analyses, there was no difference in time to virologic suppression between 2‐DRs and 3‐DRs among patients who were viraemic at regimen initiation. In unadjusted analyses, differences in time to virologic failure among patients who were virologically stable at regimen initiation was marginal between groups, but there was no difference in adjusted analyses. In both adjusted and unadjusted analyses, the time to discontinuation was faster for patients initiating a 2‐DR than patients initiating a 3‐DR.

Patients initiating a 2‐DR differed significantly at baseline compared to patients initiating a 3‐DR. Patients prescribed 2‐DRs were older and had a longer treatment duration compared to patients prescribed 3‐DRs. We also observed that patients on a 2‐DR were more likely to have diagnoses of comorbid conditions, a lower CD4 count, and a history of AIDS compared to patients taking 3‐DRs. These findings may suggest that clinicians select 2‐DRs for patients who present with a more complex treatment history. In this case, a 2‐DR could increase patient adherence and tolerability, which is especially vital for patients with known multidrug resistance. Of note, 54% of patients taking a 2‐DR were virologically stable at regimen initiation, and there could be different factors resulting in 2‐DR initiation among these patients compared to patients who were viraemic at regimen initiation.

In OPERA, there was no association between regimen type and time to virologic suppression among patients who were viraemic at regimen initiation. Previous trials have not evaluated suppression among viraemic patients, but have assessed the difference in maintenance of suppression between 2‐DR and 3‐DR groups 16, 17, 18, 19, 20, 21, 22, 23. In the SWORD‐1 and SWORD‐2 trials, the 48 weeks adjusted difference in suppression maintenance between the 2‐DR (DTG + RPV) and the 3‐DR arms was −0.2% (95% CI: −3.0, 2.5) 22, 23. In the SALT study, the difference in the proportion of patients with VL < 50 copies/mL between the 2‐DR (ATV/r + 3TC) and the 3‐DR (ATV/r + 2 NRTIs) arms was 5.7% (95% CI, −4.5%, 15.9%) at 48 weeks and 0.5% (95% CI: −9.9%, 11.0%) at 96 weeks 16. No difference in suppression maintenance was reported in two smaller trials. In one, 92% of patients on LPV/r + RAL and 88% of patients on a regimen consisting of a core agent with at least two NRTIs had a VL < 50 copies/mL at 48 weeks 19. In the other, the difference in the proportion of patients with HIV RNA < 50 copies/mL between DRV/r + RAL and LPV/r + TDF + FTC at 48 weeks was −11% (95% CI: −25%, 4%) 18. However, the AtLaS‐M study reported a statistically significant difference between the 2‐DR (ATV/r + 3TC) and the 3‐DR (ATV/r + 2 NRTIs) arms of 9.8% (95% CI: 1.2, 18.4)] 15.

Among patients who were virologically stable at regimen initiation, the association between regimen type and virologic failure was not statistically significant after adjustment for confounders. However, the point estimate remained elevated, suggesting that a lower drug count could potentially increase the risk of failure. In the AtLaS‐M study, 1.5% of 2‐DR (2/133) and 4.5% of 3‐DR patients (6/133) patients experienced virologic failure, with a difference of −3% (95% CI: −7.1, 1.1) 13. In the SALT study, 7.5% of 2‐DR (10/133) and 5.2% of 3‐DR patients (7/134) had a detectable VL at 96 weeks, but no statistical comparison was performed 16. In the RALAM study, 96% (n = 47) of the 3TC/RAL group and 80% (n = 20) of 3‐DR group remained treatment failure free (estimated difference = 0.159, 95% CI: 0.012, 0.353) at 24 weeks 21.

In this observational setting, time to discontinuation was faster for patients initiating a 2‐DR compared to patients initiating a 3‐DR. It is plausible that 2‐DRs were used as a temporary strategy to handle challenges with tolerability or to avoid drug‐drug interactions during an acute event, with clinicians planning to switch patients back to a more standard regimen after these issues were resolved 32. This strategy can be especially appealing since treatment interruptions are not recommended 33. However, clinical rationale for discontinuation of a drug is typically not well‐documented in medicals records, especially in the absence of virologic failure. Therefore, unlike clinical trials, this study could not distinguish between discontinuations prompted by adverse events, intolerances, comorbidities or other reasons.

The pooled analysis of the SWORD‐1 and SWORD‐2 trials found that patients switching to DTG + RPV were more likely to report adverse events leading to withdrawal (3% vs. <1%) than patients continuing with their current 3‐DR consisting of two NRTIs plus a third core agent (NNRTI, INSTI or PI) 22. The AtLaS‐M and SALT trials found that discontinuations due to adverse events were rare and there was no statistical difference between 2‐DR and 3‐DR groups 15, 16.

This study was conducted in a large sample of PLHIV in clinical care across the United States The diverse population provided key information on 2‐DR prescriptions in a real‐world setting. The use of electronic health records contributed accurate and detailed information on virologic outcomes and important patient characteristics. This analysis, therefore, represents a complete picture of 2‐DR prescription practices and effectiveness before the approval of the first 2‐DR regimen.

These findings are not without limitations. First, although all models were adjusted for important confounders, unmeasured and residual confounding could have biased our results. Second, it is possible that the observed differences in discontinuation between patients taking a 2‐DR and patients taking a 3‐DR could have resulted in selection bias, which would influence estimates comparing virologic suppression and virologic failure. For these data, the median time on the current regimen of interest was 12 months (IQR: 4, 24) for 2‐DR patients and 19 months (IQR: 12, 33) for 3‐DR patients. Given the shorter duration of follow‐up, it is possible there was not enough time to observe potential failure outcomes among 2‐DR patients. However, while this selection bias could have impacted the virologic suppression and failure analyses, the description of 2‐DR use in a real‐world setting, as well as the association between regimen type and time to discontinuation would not have been affected by such bias. Third, it is possible that differences in testing frequencies of VL measurements between 2‐DR and 3‐DR patients could have also resulted in bias. In the OPERA cohort, all laboratory tests are performed as part of routine clinical care at the discretion of the health care provider. The proportion of patients with at least one VL test performed over follow‐up was lower among patients taking a 2‐DR (84%) than a 3‐DR (88%, *p* = 0.01). Also, this analysis focused on any 2‐DRs and 3‐DRs initiated in OPERA and no conclusions can be reached with regards to the clinical effectiveness of specific drug combinations. Given that previous trials have assessed the effectiveness of specific 2‐DRs, these data cannot be directly compared to results from those studies. In addition, the relative proportion of patients on specific 2‐DRs with varying effectiveness could have impacted the results of this study.

Finally, our population is quite heterogeneous. Patients prescribed 2‐DRs were identified first then patients without 2‐DRs were considered if they had an eligible 3‐DR in the same time window. This strategy selected 3‐DRs earlier in their treatment journey than 2‐DRs. Regimens could have resulted from a variety of scenarios. There may be bias introduced when comparing a regimen that was selected versus a regimen that resulted due to other extenuating circumstances. We made every effort to exclude patients who were participating in clinical trials (n = 1927) as they tend to differ significantly from the general population. The presence of these uniquely compliant patients in either arm could have significantly influenced the outcome.

## 5 Conclusions

These findings demonstrate that even prior to the recent approval of the first two‐drug co‐formulations, 2‐DRs were being used among treatment‐experienced patients in US clinical practice. Virologic suppression among patients who were viraemic at regimen initiation and virologic failure among those who were virologically stable at regimen initiation were comparable for 2‐DRs and 3‐DRs. While 2‐DRs appeared to be virologically effective, discontinuation was more likely with a 2‐DR. The findings from this study suggest that 2‐DRs may be a viable alternative to 3‐DRs for ART‐experienced patients with comorbidities. Continued evaluation of specific 2‐DRs in observational settings is needed to elucidate the long‐term effectiveness of this treatment strategy in the real world.

## Competing interests

This work was supported by a project grant from ViiV Healthcare, which was performed using Epividian's OPERA® database. ViiV Healthcare had no editorial control in the conduct of this study or the content of this article. This research was a collaboration in which the OPERA Advisory Board was the final arbiter for decision making. GP is employed by Whole Family Health Center. CH was employed by Epividian when this analysis was conducted and now is employed by ViiV Healthcare. JF and GF are employed by Epividian. VV, MA and LR are employed by ViiV Healthcare.

## Authors' contributions

GP, CH and JF share the responsibility for the design of this study. CH, JF, VV, LR and GF contributed to the implementation of the study. CH and JF conducted all the analyses. GP, CH, JF, VV, MA, LR and GF contributed to the interpretation of results. JF drafted the manuscript. All authors have critically reviewed and approved the manuscript and have participated sufficiently in the work to take public responsibility for its content.

## Supporting information


**Table S1.** Most frequent 2‐DR and 3‐DR* regimens prescribed to patients treatment‐experienced, not suppressed at baseline
