# Supplementary material for: Two‐drug antiretroviral regimens: an assessment of virologic response and durability among treatment‐experienced persons living with HIV in the OPERA® Observational Database
Source: J Int AIDS Soc. 2019 Dec 4;22(12):e25418. doi: 10.1002/jia2.25418 (PMC6893210; doi:10.1002/jia2.25418)
Supplement: Supplementary file 1 — Table S1. Most frequent 2‐DR and 3‐DR* regimens prescribed to patients treatment‐experienced, not suppressed at baseline [file JIA2-22-e25418-s001.docx]

**Supplemental Table 1. Most frequent 2-DR and 3-DR* regimens prescribed to patients treatment-experienced, not suppressed at baseline**

|  | 2-DR | | 3-DR | |
| --- | --- | --- | --- | --- |
|  | Regimen | N(%) | Regimen | N(%) |
| 1 | darunavir/raltegravir | 219 (35.7%) | darunavir/emtricitabine/tenofovir | 603 (16.9%) |
| 2 | darunavir/dolutegravir | 105 (17.1%) | efavirenz/emtricitabine/tenofovir | 396 (11.1%) |
| 3 | darunavir/etravirine | 50 (8.2%) | atazanavir/emtricitabine/tenofovir | 353 (9.9%) |
| 4 | darunavir/tenofovir | 34 (5.5%) | elvitegravir/emtricitabine/tenofovir | 300 (8.4%) |
| 5 | atazanavir/tenofovir | 27 (4.4%) | abacavir/dolutegravir/lamivudine | 278 (7.8%) |
| 6 | etravirine/raltegravir | 25 (4.1%) | emtricitabine/raltegravir/tenofovir | 228 (6.4%) |
| 7 | atazanavir/raltegravir | 24 (3.9%) | emtricitabine/rilpivirine/tenofovir | 190 (5.3%) |
| 8 | lopinavir/raltegravir | 14 (2.3%) | abacavir/atazanavir/lamivudine | 137 (3.8%) |
| 9 | dolutegravir/rilpivirine | 9 (1.5%) | abacavir/darunavir/lamivudine | 126 (3.5%) |
| 10 | atazanavir/dolutegravir | 6 (1.0%) | dolutegravir/emtricitabine/tenofovir | 90 (2.5%) |

*Boosting agents were not counted when determining if a regimen was 2-DR or 3-DR
